# Mycobacterial Spindle Cell Pseudotumor (MSP): A Case Report

**DOI:** 10.7759/cureus.40432

**Published:** 2023-06-14

**Authors:** Anita Arthur, Julia C Fortier, Vladimir Vincek, Rodrigo H Valdes Rodriguez

**Affiliations:** 1 Dermatology, University of Florida, Gainesville, USA

**Keywords:** immunocompromised, hiv, derm path, spindle cell, mycobacterial

## Abstract

Mycobacterial spindle cell pseudotumor (MSP) is a rare proliferation of spindle-shaped histiocytes that occurs most frequently in lymph nodes but has also been documented in the skin, soft tissue, abdomen, and other sites. These lesions contain acid-fast mycobacteria, most commonly *Mycobacterium avium *complex. Fewer than 10 cases of cutaneous MSPs have been published, and most have occurred in immunocompromised patients, either due to human immunodeficiency virus (HIV) infection or immunosuppressive medications. The differential diagnosis includes Kaposi’s sarcoma and other spindle cell neoplasms, which can be distinguished based on histology and special stains. We present the case of a 76-year-old man with HIV infection who presented with a diffuse rash on his arms and legs. A pretibial biopsy was performed and revealed tubercular MSP.

## Introduction

Mycobacterial spindle cell pseudotumor (MSP) is a rare condition characterized by the proliferation of spindle-shaped histiocytes containing numerous mycobacterial organisms. It has been reported most commonly in lymph nodes but has also been seen in the skin, soft tissue, abdomen, lungs, nasal septum, brain, and bone marrow. It presents most frequently in immunocompromised hosts, particularly those with human immunodeficiency virus (HIV) infection [1]. To our knowledge, only eight cases involving the skin have been reported in the literature [2-9]. Herein, we report the case of a patient with HIV with a cutaneous tubercular MSP.

## Case presentation

**Figure 1:**
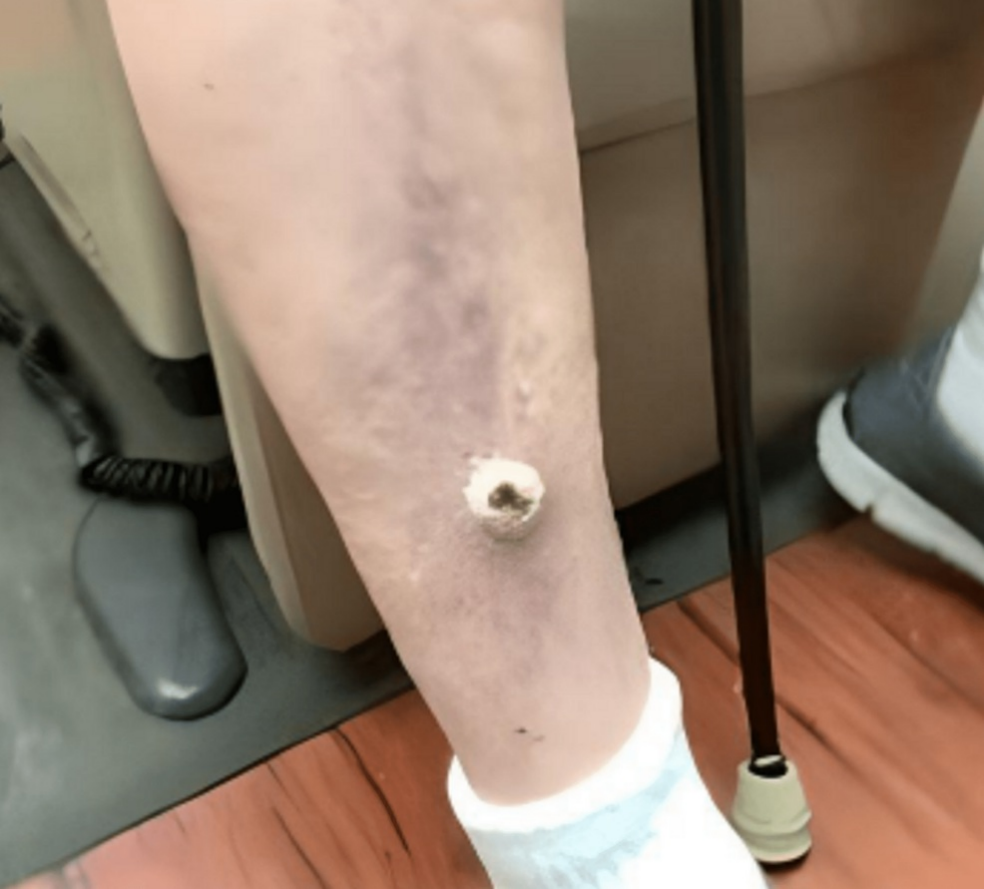
Nodule on presentation. A 2-cm erythematous tender nodule with hyperkeratotic scale over his right distal pretibial region.

The patient is a 76-year-old male with dementia and HIV infection (CD4 count of 80 cells/μL) who presented to the dermatology clinic with a two-week history of a new rash over his legs and arms. He was first diagnosed with HIV 15 years prior to presentation. He was prescribed bictegravir, tenofovir, alafenamide, and emtricitabine. He lived in an assisted living facility and his caretaker reported poor patient compliance with these medications. On physical exam, he was noted to have a 2 cm erythematous tender nodule with a hyperkeratotic scale over his right distal pretibial region (Figure 1).

**Figure 2:**
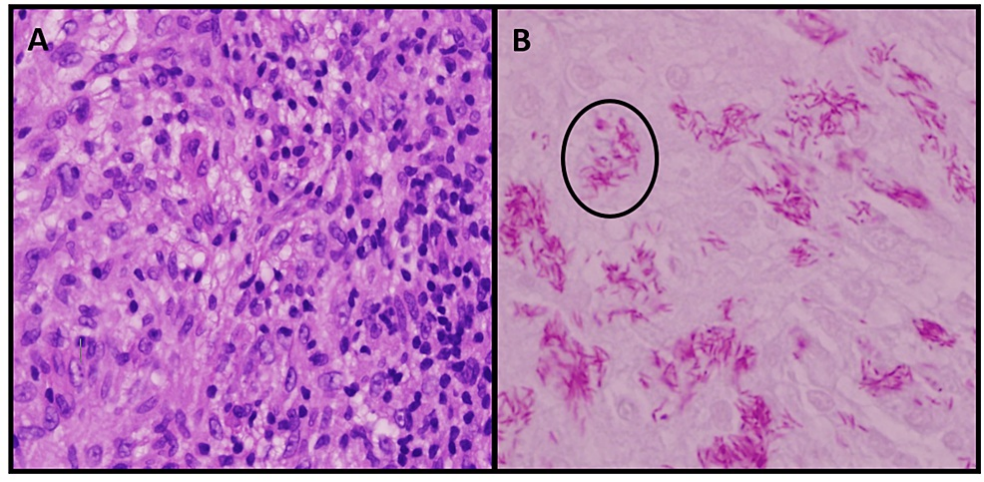
Dermatopathology of MSP. A. Hematoxylin and eosin (H&E) stain at 400x magnification demonstrating foamy histiocytes and scattered lymphocytes. B. AFB stain at 600x magnification demonstrating clusters of elongated mycobacteria (circled) within histiocytes. MSP, mycobacterial spindle cell pseudotumor; AFB, acid-fast bacilli

Dermatopathology revealed epidermal pseudoepitheliomatous hyperplasia with dermal nodules made of a mixture of lymphocytes, histiocytes, and focal neutrophilic aggregates (Figure 2A). The acid-fast bacilli (AFB) stain was positive, confirming the presence of mycobacterium (Figure 2B). Grocott methenamine silver (GMS) and Gram stain were negative. AE1/AE3, SOX10, CD30, CD34, S100, and CD10 stains were used to exclude spindle cell neoplasms. CD68, CD3, and CD20 stains confirmed the lymphohistiocytic nature of the lesion. Tissue histology was consistent with tubercular MSP. The patient was lost to follow-up before treatment could be initiated but deceased six months following the biopsy due to respiratory complications related to COVID-19 infection. 

## Discussion

Mycobacterial spindle cell pseudotumor is a rare disease that is believed to develop in response to mycobacterial infection. A review of MSPs of the skin and other organs between 1950 and 2017 noted that 84% of affected patients were male, and nearly 50% of affected patients had HIV infection [1]. Our case of a male patient with HIV reinforces these demographic associations. The most commonly affected site was the lymph node (45%), followed by the skin and soft tissue in 20% of cases. *Mycobacterium avium* complex was the most common mycobacterium isolated from these lesions (47.1%), followed by *Mycobacterium tuberculosis* complex (16%). It has been suggested that MSP is due to a dysregulated immune response, leading to a proliferation of spindle cells, which is why immunocompromising medical conditions, including HIV infection, are risk factors [4]. Published treatment has included antimycobacterial therapy, surgical resection, antimycobacterial agents plus surgery, and no treatment at all. The best outcomes were observed in patients who received antimycobacterial therapy [1]. Though our patient deceased before receiving treatment for his MSP, antimycobacterial therapy would have been most appropriate, with or without surgery, depending on the patient's preference and functional status. 

Cutaneous MSP was first reported in 1985 by Wood et al., documenting a 54-year-old cardiac transplant patient who developed progressive swelling of his hand with nodules and linear lymph node enlargement. Hematoxylin and eosin (H&E) staining of skin abscesses suggested a spindle cell neoplasm [4]. The handful of case reports documenting MSPs of the skin published since have elucidated the differential diagnosis of this condition to include soft tissue sarcoma and Kaposi’s sarcoma, as these all share the similar histopathologic feature of spindle cells arranged in a storiform pattern. The key to distinguishing MSP from Kaposi sarcoma is the presence of multinucleated cells or foamy histiocytes in MSP, mixed with lymphocytes and occasional giant cells [1]. Conversely, Kaposi's sarcoma features elongated "slit-like" vascular spaces with spindle endothelial cells, mixed with plasma cells, few lymphocytes, and prominent red blood cell extravasation. Special stains can also be used to differentiate the two, as MSP stains are positive for AFB whereas Kaposi stains are positive for human herpesvirus-8 (HHV8). An inflammatory predominance favors the diagnosis of MSP over other spindle cell neoplasms but can be further investigated with flow cytometry and various stains [8].

## Conclusions

We report a case of cutaneous MSP in a male patient with HIV, contributing to a very limited body of literature as fewer than 10 such cases have been published. Antimycobacterial therapy with or without surgery is the best treatment for this condition, though this patient unfortunately deceased before he was able to be treated. The differential diagnosis of MSP includes Kaposi sarcoma and other spindle cell neoplasms. Tissue biopsy and special stains are required for differentiation and determination of the definitive diagnosis.
